# Redefining Biomedicine: Artificial Intelligence at the Forefront of Discovery

**DOI:** 10.3390/biom14121597

**Published:** 2024-12-14

**Authors:** Nguyen Quoc Khanh Le

**Affiliations:** 1 In-Service Master Program in Artificial Intelligence in Medicine, College of Medicine, Taipei Medical University, Taipei 110, Taiwan; khanhlee@tmu.edu.tw; Tel.: +886-2-2736-1661 (ext. 7174); 2 AIBioMed Research Group, Taipei Medical University, Taipei 110, Taiwan; 3 Translational Imaging Research Center, Taipei Medical University Hospital, Taipei 110, Taiwan

The rapid evolution of artificial intelligence (AI) is redefining biomedicine, placing itself at the forefront of groundbreaking discoveries in molecular biology, genomics, drug discovery, diagnostics, and beyond (contributions 1–3). This Special Issue of *Biomolecules* features 13 exemplary contributions that demonstrate the breadth and depth of AI applications in biomedicine, encompassing both foundational research and cutting-edge innovations.

Collectively, these contributions tackle an array of biomedical challenges, harnessing advanced AI techniques such as deep learning, graph neural networks (GNNs), and natural language processing (NLP) to drive discovery and innovation. They are organized into two main categories: original research articles and review papers. Together, these contributions illustrate AI’s transformative potential to uncover biological insights and improve clinical outcomes.

Advances in molecular and structural biology are highlighted by studies addressing challenges in protein modeling, RNA classification, and molecular property prediction. For instance, deepBBQ employs convolutional neural networks to reconstruct protein backbones from Cα coordinates with high accuracy and computational efficiency, maintaining stereochemical integrity (contribution 4). In RNA classification, ConF integrates bidirectional long short-term memory (BiLSTM), convolutional neural network (CNN), and cross multi-head attention mechanisms to achieve precise predictions for noncoding RNA families, including complex structures like pseudoknots (contribution 5). Meanwhile, DRpred combines Bayesian networks with BiLSTM and attention mechanisms to deliver multi-label predictions for mRNA subcellular localization, and mRCat employs gradient boosting and large language models for the binary classification of mRNA localization (contributions 6 and 7). Complementing these efforts, a hybrid framework in molecular property prediction combines LSTM and graph attention networks to leverage simplified molecular input line entry system (SMILES) strings and graph-based features for molecular property modeling, advancing computer-aided drug design (contribution 8). These studies collectively exemplify AI’s role in unraveling molecular complexities and enhancing biological understanding.

In drug discovery and cancer research, AI is proving pivotal in predicting drug synergies, identifying epigenetic biomarkers, and integrating multi-modal data for diagnostics. SynerGNet, a GNN model, predicts anticancer drug synergy by incorporating heterogeneous biological features into protein interaction networks, achieving superior accuracy through augmented training data (contribution 9). Similarly, G4Beacon utilizes gradient-boosting decision trees and chromatin accessibility data to predict in vivo G-quadruplex (G4) structures, critical for epigenetic regulation, across diverse cell types with remarkable precision (contribution 10). Furthermore, the integration of radiomics and serum metabolomics for lung cancer diagnosis demonstrates radiomics’ strong discriminatory power, with modest improvements observed in combined models (contribution 11). These studies illustrate AI’s transformative role in addressing complex challenges in oncology and pharmacology, offering new pathways for therapeutic development and personalized cancer care.

The integration of multi-modal data and NLP is reshaping biomedical research by enabling advanced disease classification and efficient knowledge extraction. A transformer-based multi-modal data fusion method combines GNNs for physiological and biochemical data with 3D CNNs for imaging data, utilizing cross-modal transformers to enhance feature representation and classification accuracy in chronic obstructive pulmonary disease (COPD) diagnosis (contribution 12). On the NLP front, KnowVID-19 employs machine learning and keyword extraction techniques to create a knowledge-based system for targeted COVID-19 literature retrieval (contribution 13). By structuring publication data and visualizing term networks, KnowVID-19 enhances the exploration of emerging research trends. Together, these approaches showcase the power of AI in bridging diverse data sources and extracting actionable insights, paving the way for improved diagnostics and research efficiency.

AI is also modernizing traditional and complementary medicine by enhancing the precision, interpretability, and applicability of treatment recommendations. A machine learning-based approach for herbal formulae recommendation integrates clinical data, Sasang constitutional types, and expert knowledge to predict tailored treatments in Korean medicine (contribution 14). Using a cascaded deep forest model combined with data imputation and oversampling techniques, this method achieves high accuracy even with incomplete data. The inclusion of interpretable AI techniques, such as local interpretable model-agnostic explanations (LIME), provides transparent insights into the clinical features influencing recommendations, bridging the gap between machine learning outputs and practitioner trust. This approach highlights AI’s potential to standardize and optimize traditional practices while retaining their personalized and holistic essence.

The review papers in this Special Issue provide a comprehensive exploration of foundational and emerging methodologies in protein research. One review focuses on advances in protein subcellular localization, detailing AI-based techniques—sequence-based, knowledge-based, and image-based—that address challenges in multi-label localization and provide insights into drug discovery and disease mechanisms (contribution 15). Another review examines the evolution of multiple sequence alignment (MSA), a cornerstone of molecular biology, highlighting its critical role in structure prediction and functional annotation (contribution 16). The review further explores emerging AI-driven alternatives, such as protein language models, which complement or replace traditional MSA in complex tasks, offering scalable and efficient solutions. Together, these reviews illuminate the synergy between traditional methods and AI, providing a roadmap for future advancements in structural biology and cellular function prediction.

This Special Issue underscores how AI is redefining the landscape of biomedicine, pushing the boundaries of discovery and innovation from molecular insights to clinical applications. The diverse methodologies and applications presented here reflect the interdisciplinary nature of AI, bridging computational innovation with biomedical research. As AI technologies continue to evolve, their integration into biomedicine will undoubtedly yield transformative impacts on healthcare, diagnostics, and therapeutics.

We extend our heartfelt gratitude to all authors and reviewers for their invaluable contributions, which have made this Special Issue a success. We hope this collection inspires further research and fosters collaboration across disciplines.

